# Supplementary material for: Macro-scale relationship between body mass and timing of bird migration
Source: Nat Commun. 2024 May 15;15:4111. doi: 10.1038/s41467-024-48248-7 (PMC11096376; doi:10.1038/s41467-024-48248-7)
Supplement: Supplementary file 1 — Supplementary Information [file 41467_2024_48248_MOESM1_ESM.pdf]

Supplementary Information

Macro-scale relationship between body mass and timing of bird migration

Xiaodan Wang[1,2], Marius Somveille[2], Adriaan M. Dokter[3], Wenhua Cao[1], Chuyu Cheng[1], Jiajia Liu[1], Zhijun Ma[1*]

Supplementary Fig. 1. Flowchart showing the selection process for eligible papers from published papers from the Web of Science All Databases (WoS) and Scopus databases between 1 January 1900 and 1 January 2022.

Supplementary Fig. 2. Density distribution of the timing of key events in the annual cycle of migratory birds examined in this study. The dashed lines represent the mean date of each key event.

Supplementary Fig. 3. Performance of candidate variables in explaining time allocation of migratory birds. Coefficient estimates with 95% (fine line) and 50% (thick line) credible intervals for the length of (a) spring migration, (b) breeding, (c) autumn migration and (d) non-breeding period. The coefficients indicate effect size of the standardized variables; red indicates credible intervals that exclude zero. The absolute value of the non-breeding latitude was used as the non-breeding latitude.

Supplementary Fig. 4. Relationships between body mass and time allocation of migratory birds for (a) spring migration, (b) breeding, (c) autumn migration and (d) non-breeding period. Dots represent each tracked individual or population. Light bands represent 95% credible intervals. The density distribution of each variable is plotted alongside the scatter plots.

Supplementary Fig. 5. Performance of body mass in explaining breeding latitude (a, using the combined data of birds captured at stopover and non-breeding sites), and in

explaining non-breeding latitude (b, using the data of birds captured at breeding sites). Coefficient estimates with 95% (fine line) and 50% (thick line) credible intervals for the length of (a-b). The coefficients indicate effect size of the standardized variables; red indicates credible intervals that exclude zero. The absolute value of the non-breeding latitude was used as the non-breeding latitude.

Supplementary Fig. 6. Relationships between body mass and breeding latitudes (a & b) and mean non-breeding latitudes of tracked birds (c). Dots represent each tracked individual or population for the 1708 full annual tracking data (a), and each of the 311 individual or population captured at stopover and non-breeding sites (b). Non-breeding latitudes in the Southern Hemisphere are represented by negative values. The light band represents 95% confidence interval. Dots represent each tracked species (c). The horizontal and vertical dashed lines indicate that the average latitudes of non-breeding grounds for each species weighing more than 1.1 kg (36 species) were all located in north of the equator (c). The density distribution of each variable is plotted alongside the scatter plots.

Supplementary Fig. 7. Relationships between breeding latitude (a), non-breeding latitude (b), body mass (c), and migration distance of tracked birds in structural equation models. Light bands represent 95% credible intervals. Dots represent each tracked individual or population. The density distribution of each variable is plotted alongside the scatter plot. The absolute value of the non-breeding latitude was used as the non-breeding latitude for birds wintering in the Southern Hemisphere.

Supplementary Fig. 8. Carry-over effects of annual migration timing. This figure shows the relationships between departure and arrival dates across the annual cycle of migratory birds. Light bands represent 95% credible intervals. Dots represent each tracked individual or population. The density distribution of each variable is plotted alongside the scatter plots.

Supplementary Fig. 9. Results of the structural equation model analysis on migration timing of tracked birds in Passeriformes. Arrows represent the direct effects of variables on the migration timing of birds. Red arrows represent positive significant effects, blue arrows represent negative significant effects (credible intervals without zero). R^2_m, marginal R square, represents only the variance of the fixed effects, R^2_c, conditional R square, represents both the fixed and random effects. The absolute value of the non-breeding latitude was used as the non-breeding latitude.

Supplementary Fig. 10. The standard deviation (SD) of random effect phylogenetic relationship, migration year, paper and species in the structural equation models. Coefficient estimates with 95% (fine line) and 50% (thick line) credible intervals.

Supplementary Fig. 11. Performance of candidate variables in explaining migration distance and migration timing of migratory birds. Coefficient estimates with 95% (fine line) and 50% (thick line) credible intervals. The coefficients indicate the effect size of the standardized variables; red indicates credible intervals that exclude zero. The absolute value of the non-breeding latitude was used as the non-breeding latitude.

Supplementary Fig. 12. Results of the structural equation model analysis considering the effect of sex on migration timing. Arrows represent the direct effects of variables on the migration timing of birds. Red arrows represent positive significant effects, and blue arrows represent negative significant effects (credible intervals without zero). The dashed line arrows represent non-significant relationships (credible intervals with zero). R^2_m, marginal R square, represents only the variance of the fixed effects, R^2_c, conditional R square, represents both the fixed and random effects. The absolute value of the non-breeding latitude was used as the non-breeding latitude.

Supplementary Fig. 13. Arrival dates of female and male migratory birds at breeding sites. Bars show the 95% credible intervals).

Supplementary Fig. 14. The frequency distribution of body mass of all migratory species in the world (green, data from Tobias et al., 2022) and of 186 migratory species in this study (blue).

Supplementary Fig. 15. Relationships between migration distances and non-breeding latitudes of tracked birds. The latitudes of the Southern Hemisphere are represented by negative values. The light band represents 95% credible interval. Dots represent each tracked individual or population. The density distribution of each variable is plotted alongside the scatter plot.

Supplementary Fig. 16. The posterior predictive checks for departure date from non-breeding site (a), arrival date at breeding site (b), departure date from breeding site (c), arrival date at non-breeding site (d), breeding latitude (e), non-breeding latitude (f) and migration distance (g).

Supplementary Table 1. The numbers of tracked individuals, species, and published papers in each order in this study.

Supplementary Table 2. Standardized direct effects, indirect effects, and total effects between variables and four migration timing among migrant species taking sex into account estimated by structural equation model analysis.

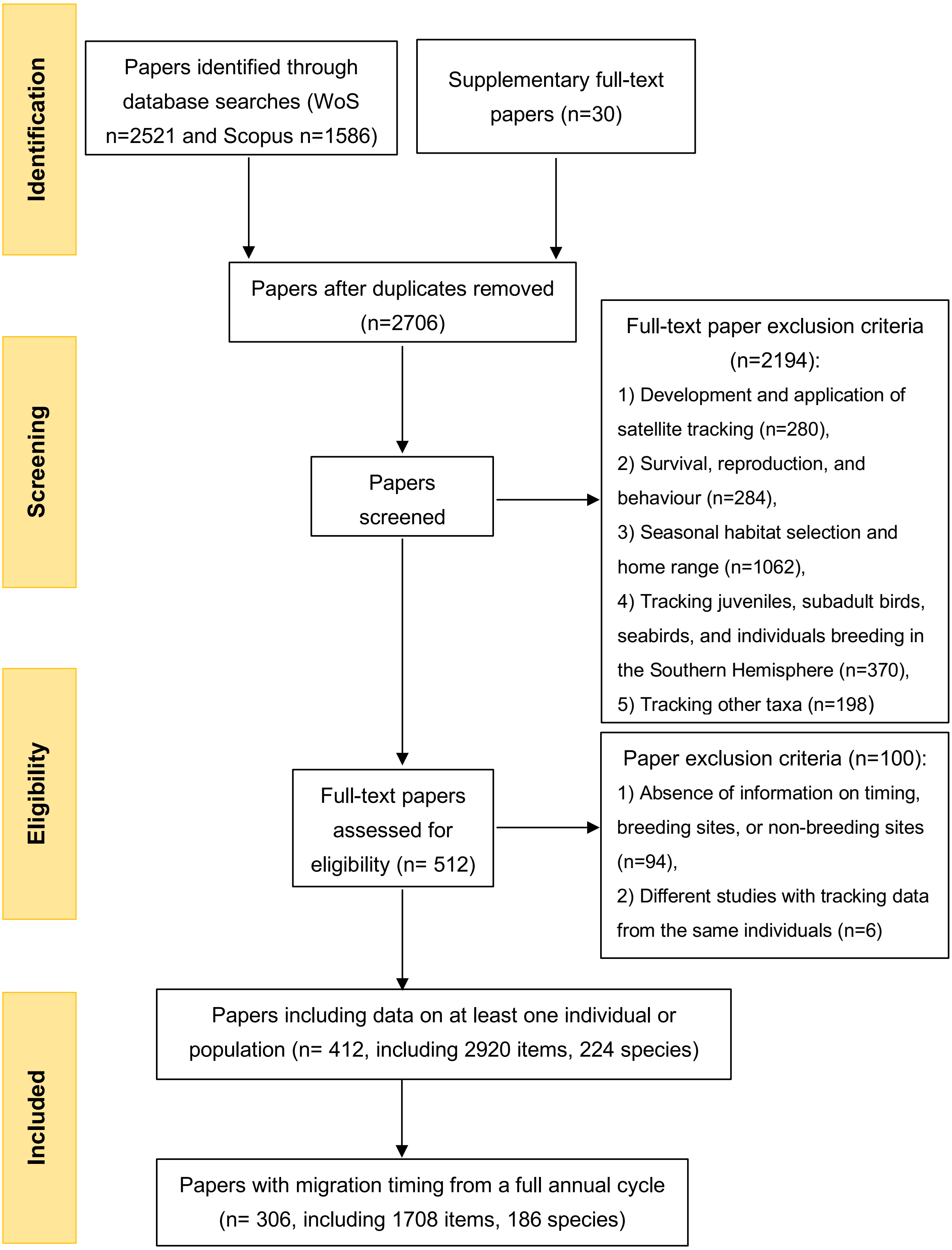

Supplementary Fig. 1. Flowchart showing the selection process for eligible papers from published papers from the Web of Science All Databases (WoS) and Scopus databases between 1 January 1900 and 1 January 2022.

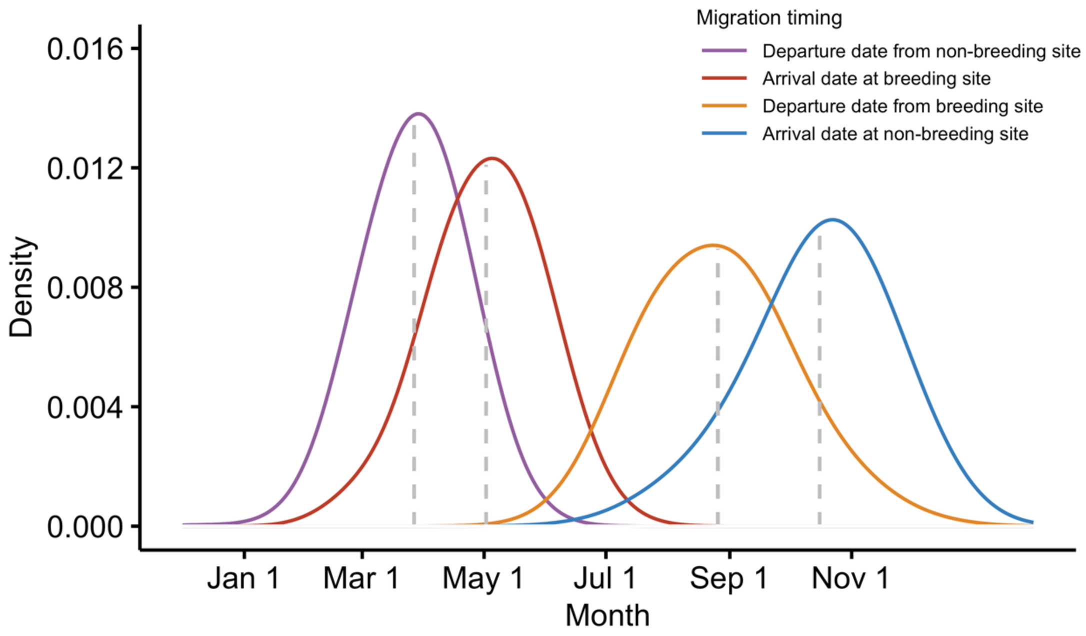

Supplementary Fig. 2. Density distribution of the timing of key events in the annual cycle of migratory birds examined in this study. The dashed lines represent the mean date of each key event.

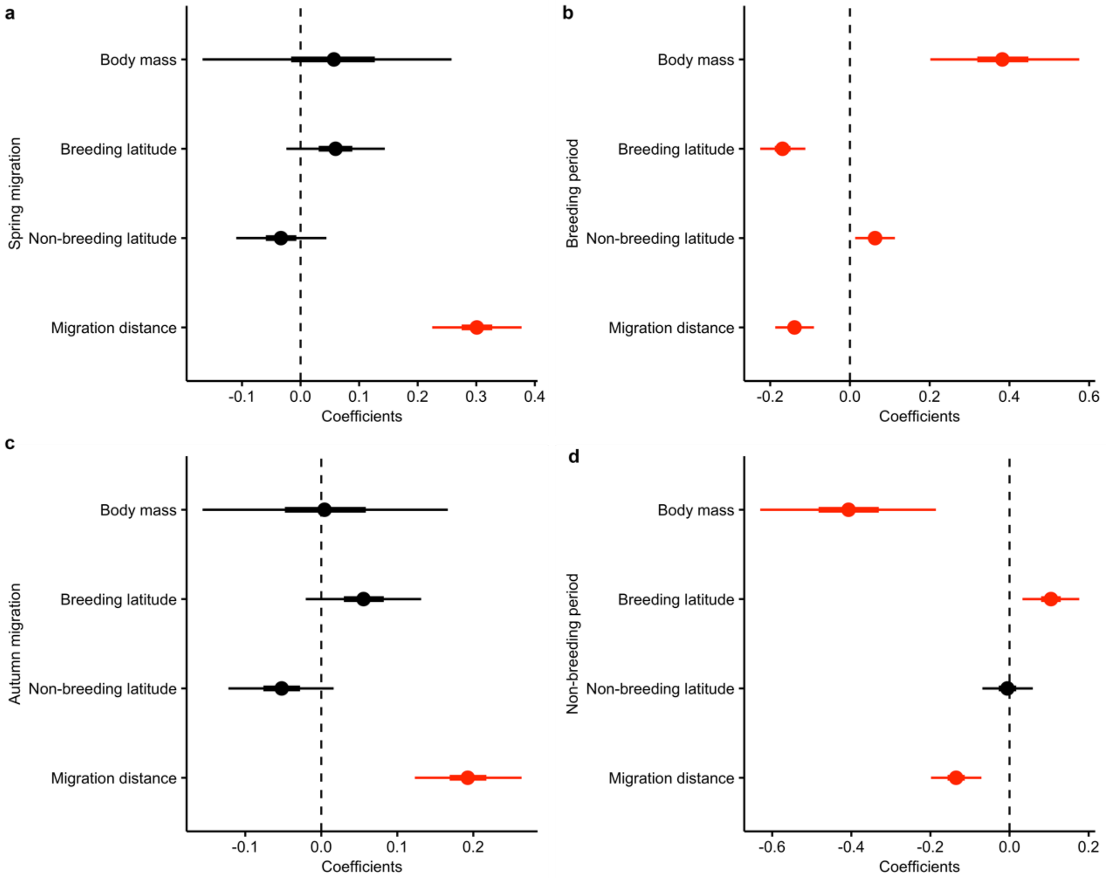

Supplementary Fig. 3. Performance of candidate variables in explaining time allocation of migratory birds. Coefficient estimates with 95% (fine line) and 50% (thick line) credible intervals for the length of (a) spring migration, (b) breeding, (c) autumn migration and (d) non-breeding period. The coefficients indicate effect size of the standardized variables; red indicates credible intervals that exclude zero. The absolute value of the non-breeding latitude was used as the non-breeding latitude.

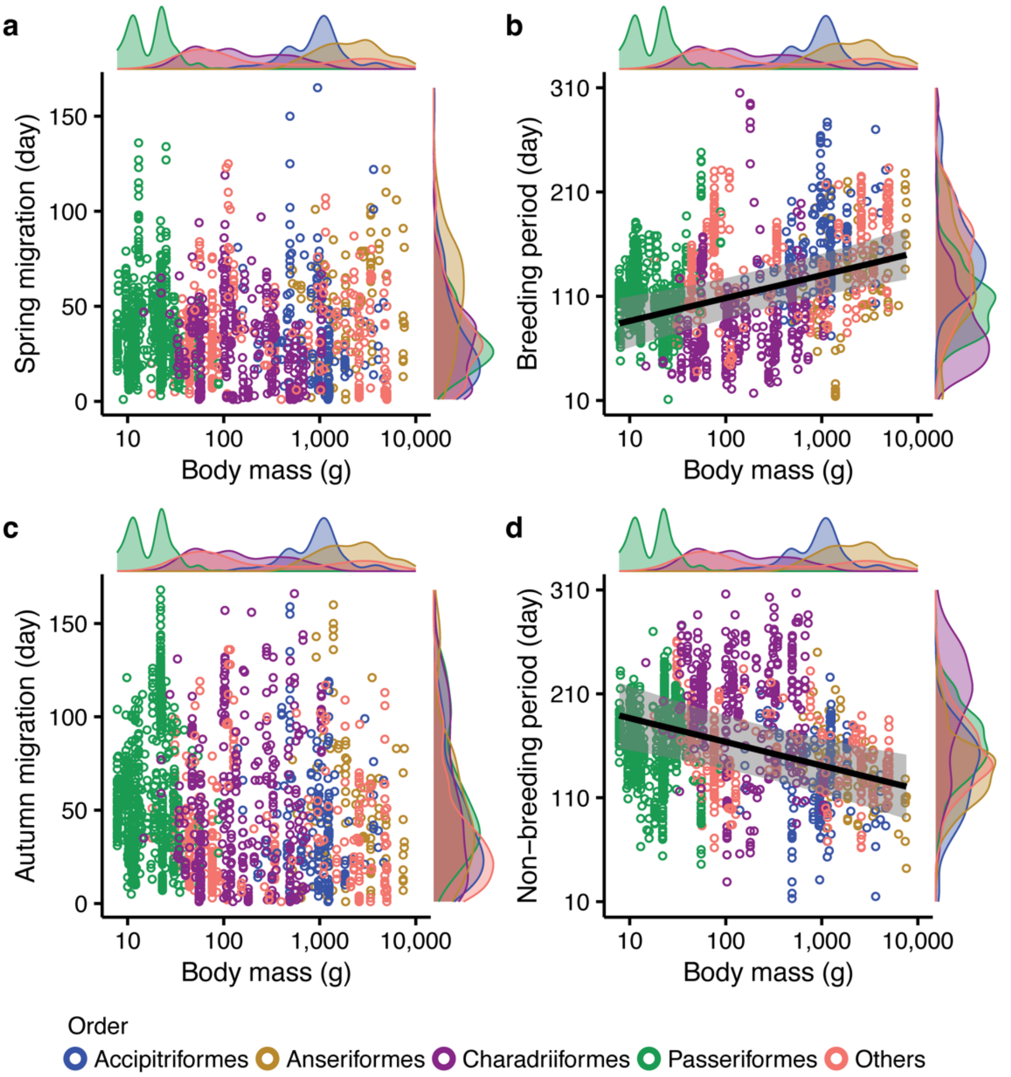

Supplementary Fig. 4. Relationships between body mass and time allocation of migratory birds for (a) spring migration, (b) breeding, (c) autumn migration and (d) non-breeding period. Dots represent each tracked individual or population. Light bands represent 95% credible intervals. The density distribution of each variable is plotted alongside the scatter plots.

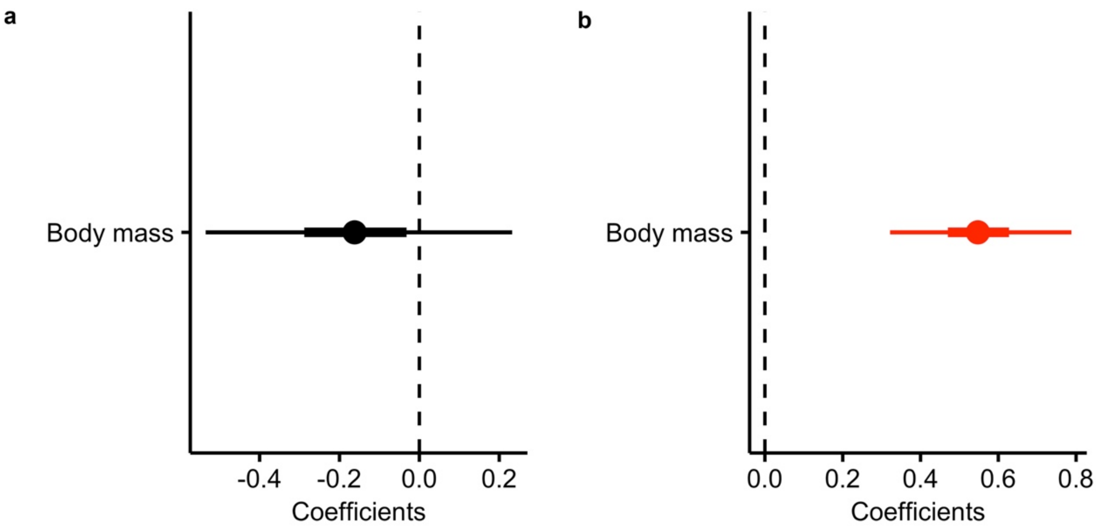

Supplementary Fig. 5. Performance of body mass in explaining breeding latitude (a, using the combined data of birds captured at stopover and non-breeding sites), and in explaining non-breeding latitude (b, using the data of birds captured at breeding sites). Coefficient estimates with 95% (fine line) and 50% (thick line) credible intervals for the length of (a-b). The coefficients indicate effect size of the standardized variables; red indicates credible intervals that exclude zero. The absolute value of the non-breeding latitude was used as the non-breeding latitude.

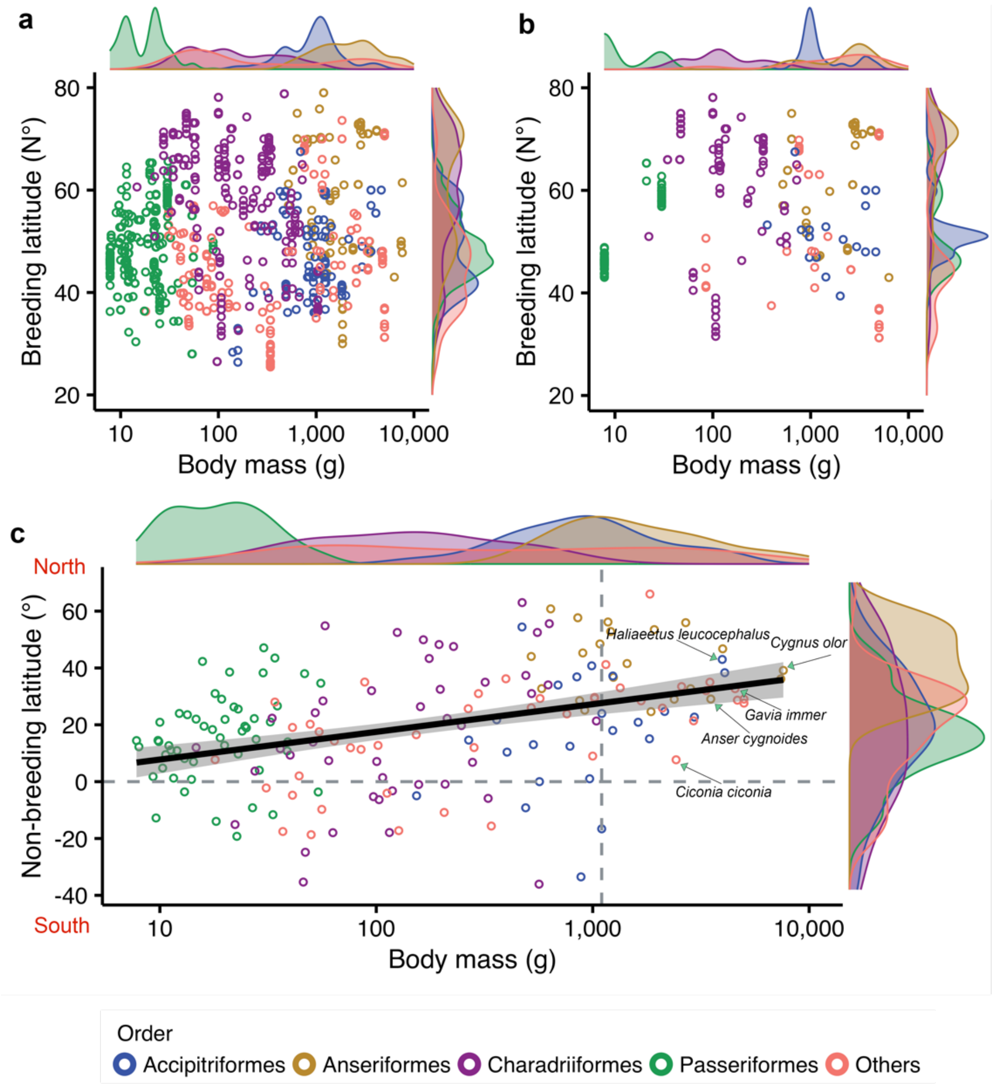

Supplementary Fig. 6. Relationships between body mass and breeding latitudes (a & b) and mean non-breeding latitudes of tracked birds (c). Dots represent each tracked individual or population of the 1708 full annual tracking data (a), and each of the 311 individual or population captured at stopover and non-breeding sites (b). Non-breeding latitudes in the Southern Hemisphere are represented by negative values. The light band represents 95% confidence interval. Dots represent each tracked species (c). The horizontal and vertical dashed lines indicate that the average latitudes of non-breeding grounds for each species weighing more than 1.1 kg (36 species) were all located in north of the equator (c). The density distribution of each variable is plotted alongside the scatter plots.

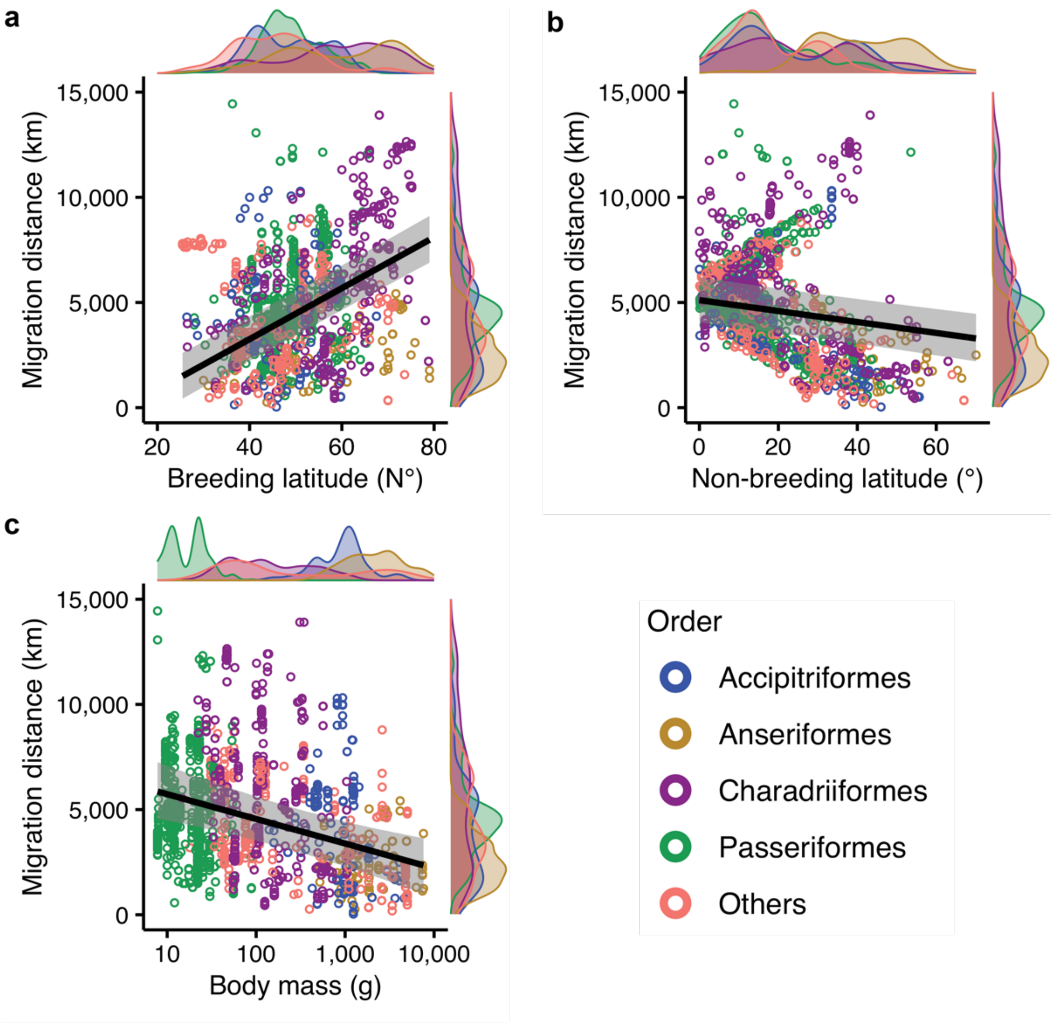

Supplementary Fig. 7. Relationships between breeding latitude (a), non-breeding latitude (b), body mass (c), and migration distance of tracked birds in structural equation models. Light bands represent 95% credible intervals. Dots represent each tracked individual or population. The density distribution of each variable is plotted alongside the scatter plot. The absolute value of the non-breeding latitude was used as the non-breeding latitude for birds wintering in the Southern Hemisphere.

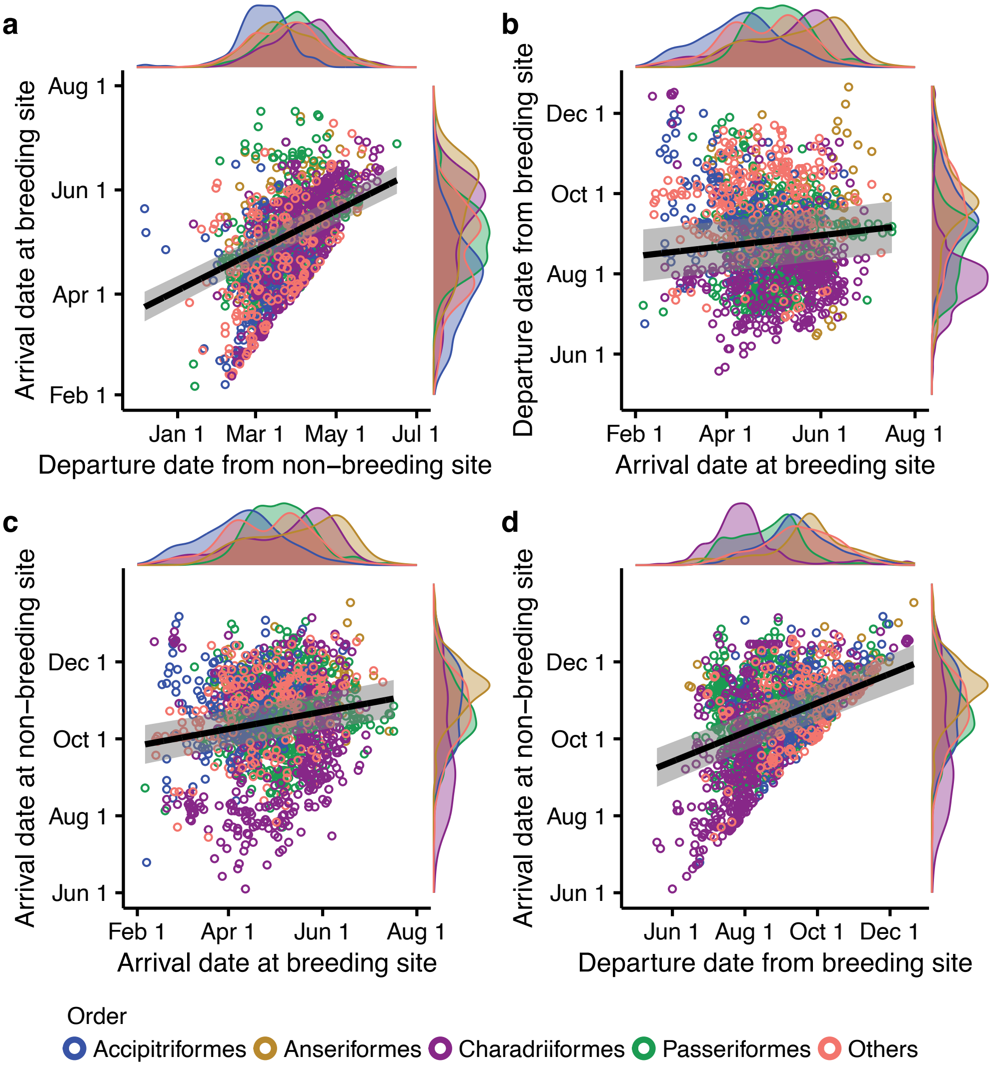

Supplementary Fig.8. Carry-over effects of annual migration timing. This figure shows the relationships between departure and arrival dates across the annual cycle of migratory birds. Light bands represent 95% credible intervals. Dots represent each tracked individual or population. The density distribution of each variable is plotted alongside the scatter plots.

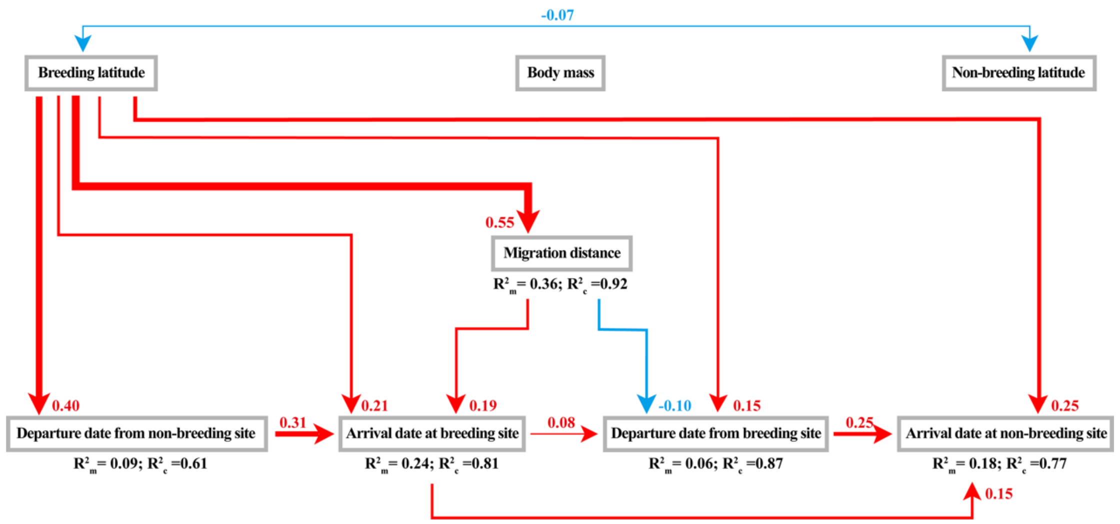

Supplementary Fig. 9. Results of the structural equation model analysis on migration timing of tracked birds in Passeriformes. Arrows represent the direct effects of variables on the migration timing of birds. Red arrows represent positive significant effects, blue arrows represent negative significant effects (credible intervals without zero). R^2_m, marginal R square, represents only the variance of the fixed effects, R^2_c, conditional R square, represents both the fixed and random effects. The absolute value of the non-breeding latitude was used as the non-breeding latitude.

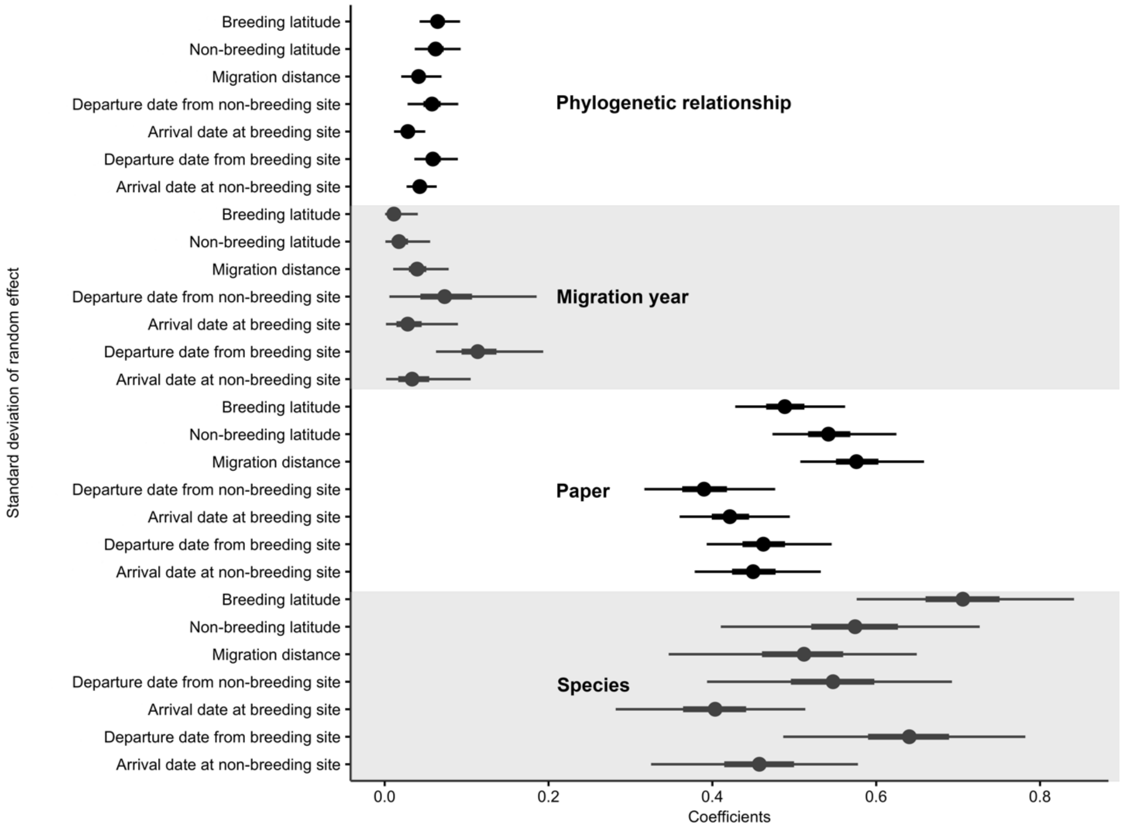

Supplementary Fig. 10. The standard deviation (SD) of random effect of phylogenetic relationship, migration year, paper and species in structural equation models.

Coefficient estimates with 95% (fine line) and 50% (thick line) credible intervals.

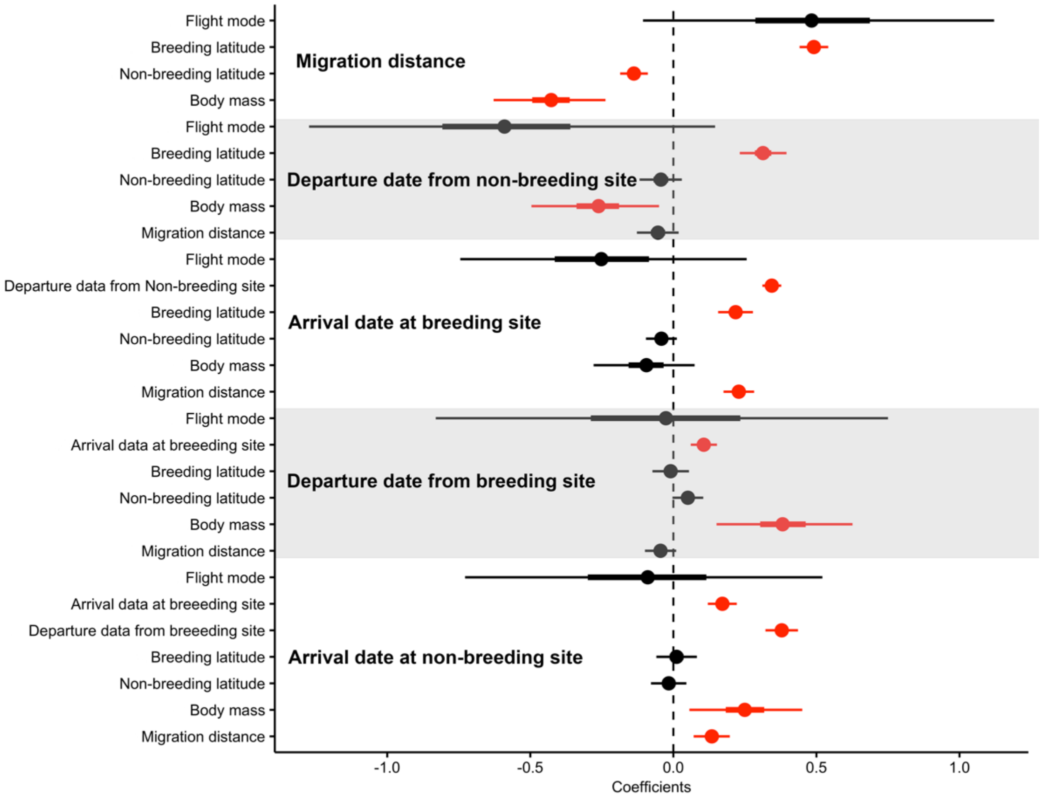

Supplementary Fig. 11. Performance of candidate variables in explaining migration distance and migration timing of migratory birds. Coefficient estimates with 95% (fine line) and 50% (thick line) credible intervals. The coefficients indicate the effect size of the standardized variables; red indicates credible intervals that exclude zero. The absolute value of the non-breeding latitude was used as the non-breeding latitude.

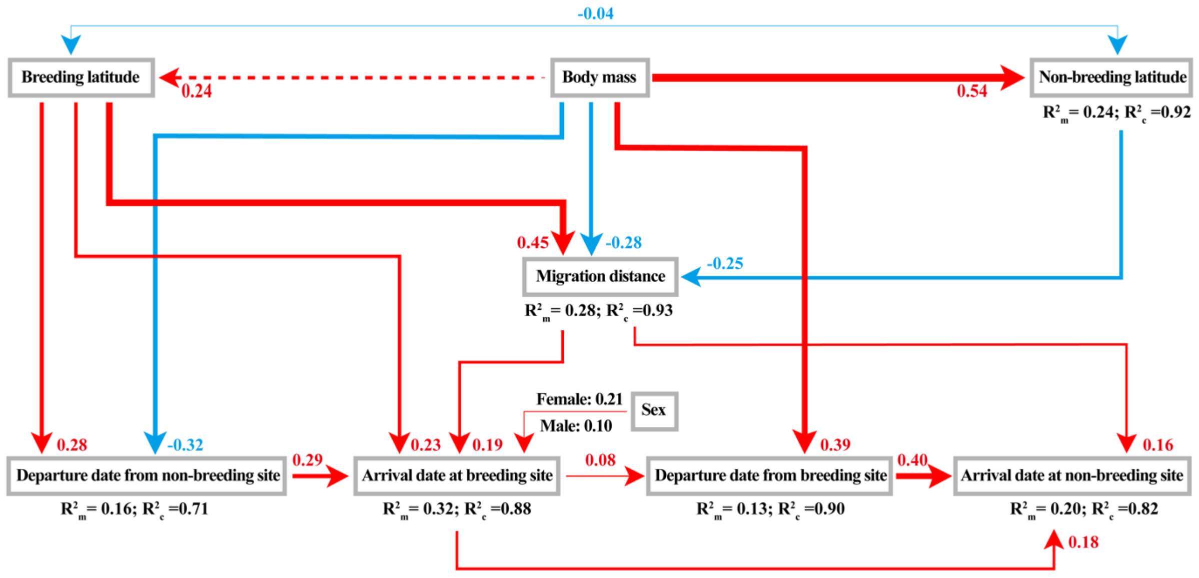

Supplementary Fig. 12. Results of the structural equation model analysis considering the effect of sex on migration timing. Arrows represent the direct effects of variables on the migration timing of birds. Red arrows represent positive significant effects, and blue arrows represent negative significant effects (credible intervals without zero). The dashed line arrows represent non-significant relationships (credible intervals with zero). R^2_m, marginal R square, represents only the variance of the fixed effects, R^2_c, conditional R square, represents both the fixed and random effects. The absolute value of the non-breeding latitude was used as the non-breeding latitude.

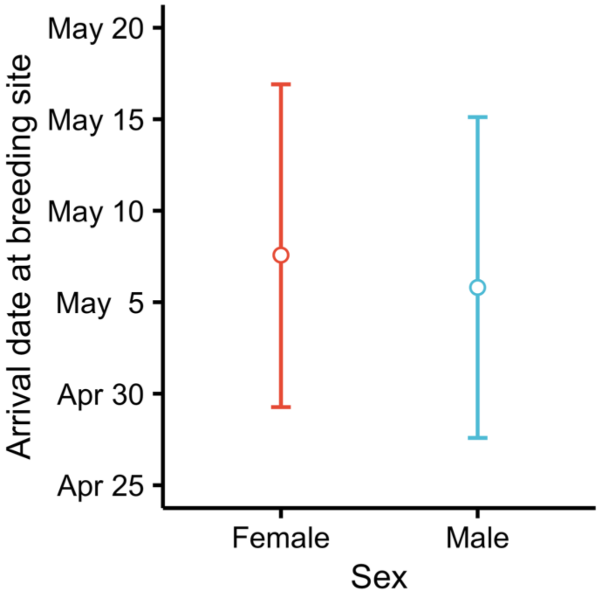

Supplementary Fig. 13. Arrival dates of female and male migratory birds at breeding sites. Bars show the 95% credible intervals).

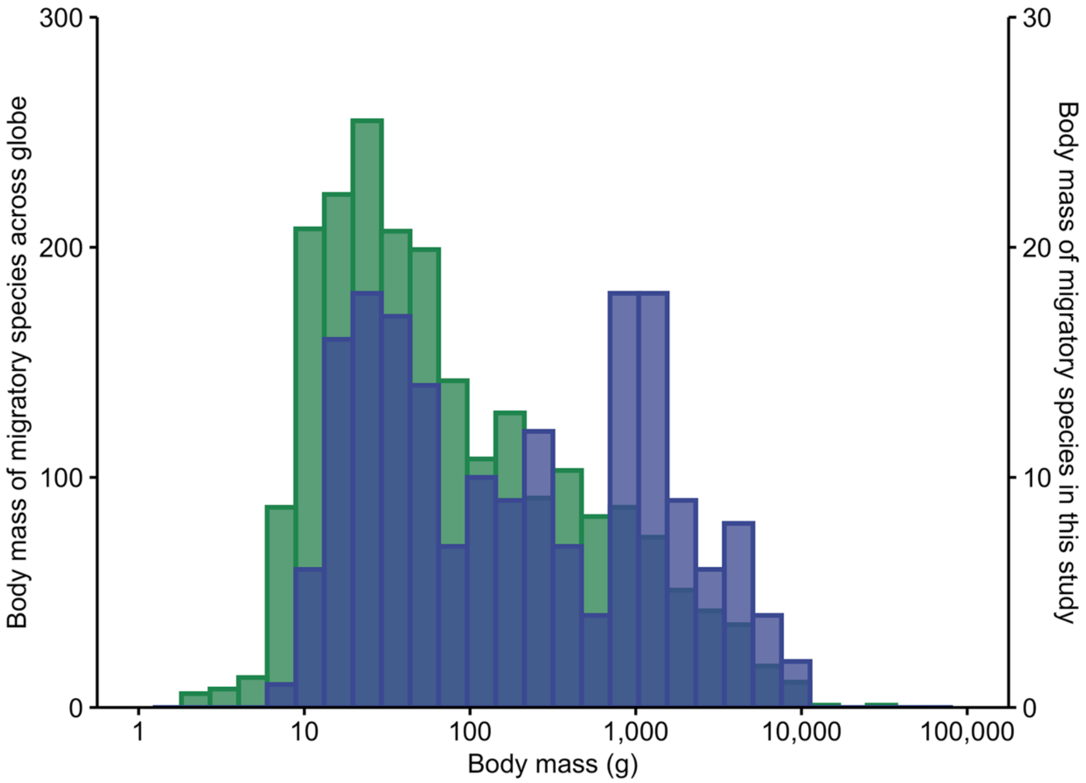

Supplementary Fig. 14. The frequency distribution of body mass of all migratory species in the world (green, data from Tobias et al., 2022) and of 186 migratory species in this study (blue).

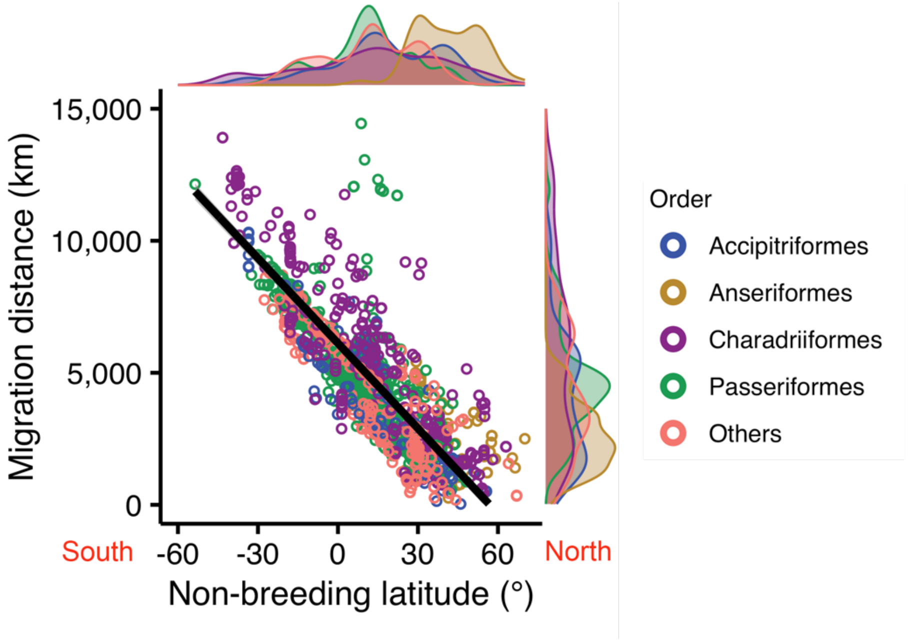

Supplementary Fig. 15. Relationships between migration distances and non-breeding latitudes of tracked birds. The latitudes of the Southern Hemisphere are represented by negative values. The light band represents 95% credible interval. Dots represent each tracked individual or population. The density distribution of each variable is plotted alongside the scatter plot.

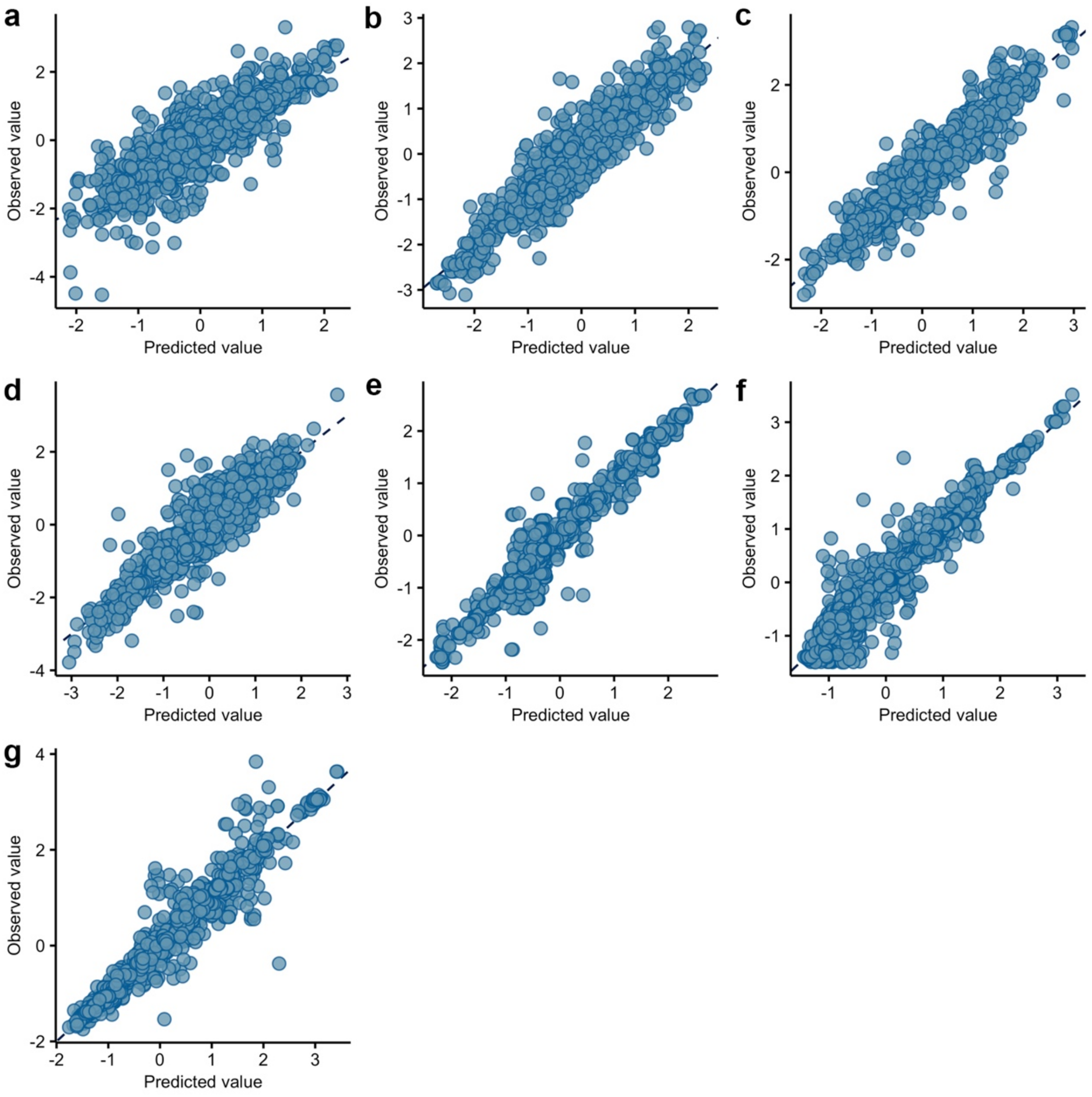

Supplementary Fig. 16. The posterior predictive checks for departure date from non-breeding site (a), arrival date at breeding site (b), departure date from breeding site (c), arrival date at non-breeding site (d), breeding latitude (e), non-breeding latitude (f), and migration distance (g).

Supplementary Table 1. The numbers of tracked individuals, species, and published papers in each order in the study.

Order	Number of tracked individuals	Number of tracked species	Number of published papers
Passeriformes	680	53	78
Charadriiformes	388	44	84
Accipitriformes	179	24	40
Anseriformes	100	21	39
Apodiformes	91	5	9
Coraciiformes	57	2	5
Gruiformes	53	8	12
Falconiformes	40	4	7
Gaviiformes	24	2	3
Ciconiiformes	15	2	5
Otidiformes	15	3	5
Strigiformes	15	3	4
Cuculiformes	14	3	5
Pelecaniformes	12	6	6
Caprimulgiformes	9	2	5
Columbiformes	8	2	3
Bucerotiformes	5	1	2
Suliformes	2	1	1
Piciformes	1	1	1

Supplementary Table 2. Standardized direct effects, indirect effects, and total effects between variables and four migration timing among migrant species taking sex into account estimated by structural equation model analysis.

		Explanatory variables							
Timing	Effect	Body mass	Breeding latitude	Non-breeding latitude	Migration distance	Sex	Departure date from non-breeding site	Arrival date at breeding site	Departure date from breeding site
Departure date from non-breeding site	Direct	-0.32	0.28	-	-	-	-	-	-
	Indirect	-	-	-	-	-	-	-	-
	Total	-0.32	0.28	-	-	-	-	-	-
Arrival date at breeding site	Direct	-	0.23	-	0.19	-0.06	0.29	-	-
	Indirect	-0.17	0.17	-0.05	-	-	-	-	-
	Total	-0.17	0.40	-0.05	0.19	-0.06	0.29	-	-
Departure date from breeding site	Direct	0.39	-	-	-	-	-	0.08	-
	Indirect	-0.01	0.03	-0.00	0.02	-	0.02	-	-
	Total	0.38	0.03	-0.00	0.02	-	0.02	0.08	-
Arrival date at non-breeding site	Direct	-	-	-	0.16	-	0	0.18	0.40
	Indirect	0.05	0.16	-0.05	0.04	-	0.06	0.03	-
	Total	0.05	0.16	-0.05	0.20	-	0.06	0.21	0.40
