# Supplementary material for: Macro-scale relationship between body mass and timing of bird migration
Source: Nat Commun. 2024 May 15;15:4111. doi: 10.1038/s41467-024-48248-7 (PMC11096376; doi:10.1038/s41467-024-48248-7)
Supplement: Supplementary file 3 — Description of Additional Supplementary Files [file 41467_2024_48248_MOESM3_ESM.pdf]

Description of Additional Supplementary Files

File Name: Supplementary Data 1

Description: Lists of migratory species with full annual migration data and the sources of data that are involved in this study.
