# Supplementary material for: Macro-scale relationship between body mass and timing of bird migration
Source: Nat Commun. 2024 May 15;15:4111. doi: 10.1038/s41467-024-48248-7 (PMC11096376; doi:10.1038/s41467-024-48248-7)
Supplement: Supplementary file 5 — Reporting Summary [file 41467_2024_48248_MOESM5_ESM.pdf]

nature portfolio

Corresponding author(s): Zhijun Ma

Last updated by author(s): Mar 25, 2024

Reporting Summary

Nature Portfolio wishes to improve the reproducibility of the work that we publish. This form provides structure for consistency and transparency in reporting. For further information on Nature Portfolio policies, see our Editorial Policies and the Editorial Policy Checklist.

Statistics

For all statistical analyses, confirm that the following items are present in the figure legend, table legend, main text, or Methods section.

n/a	Confirmed	
☐	☒	The exact sample size (*n*) for each experimental group/condition, given as a discrete number and unit of measurement
☐	☒	A statement on whether measurements were taken from distinct samples or whether the same sample was measured repeatedly
☐	☒	The statistical test(s) used AND whether they are one- or two-sided<br>*Only common tests should be described solely by name; describe more complex techniques in the Methods section.*
☐	☒	A description of all covariates tested
☐	☒	A description of any assumptions or corrections, such as tests of normality and adjustment for multiple comparisons
☐	☒	A full description of the statistical parameters including central tendency (e.g. means) or other basic estimates (e.g. regression coefficient) AND variation (e.g. standard deviation) or associated estimates of uncertainty (e.g. confidence intervals)
☐	☒	For null hypothesis testing, the test statistic (e.g. *F*, *t*, *r*) with confidence intervals, effect sizes, degrees of freedom and *P* value noted<br>*Give P values as exact values whenever suitable.*
☐	☒	For Bayesian analysis, information on the choice of priors and Markov chain Monte Carlo settings
☐	☒	For hierarchical and complex designs, identification of the appropriate level for tests and full reporting of outcomes
☐	☒	Estimates of effect sizes (e.g. Cohen's *d*, Pearson's *r*), indicating how they were calculated

Our web collection on statistics for biologists contains articles on many of the points above.

Software and code

Policy information about availability of computer code

Data collection	We conducted eligible papers with key words that were published between 1 January 1900 and 1 January 2022 in the Web of Science (All Databases) and in Scopus, and the annual migration data of migratory species were complied from Supplementary Data 1. Body mass were obtained from Dunning 2007 (https://doi.org/10.1201/9781420064452). The phylogeny trees of migratory birds were extracted from Jetz et al 2012 (https://birdtree.org/subsets/).
Data analysis	All analysis were performed in the R software v. 4.21.1 (2022).

For manuscripts utilizing custom algorithms or software that are central to the research but not yet described in published literature, software must be made available to editors and reviewers. We strongly encourage code deposition in a community repository (e.g. GitHub). See the Nature Portfolio guidelines for submitting code & software for further information.

Data

Policy information about availability of data

All manuscripts must include a data availability statement. This statement should provide the following information, where applicable:

- Accession codes, unique identifiers, or web links for publicly available datasets
- A description of any restrictions on data availability
- For clinical datasets or third party data, please ensure that the statement adheres to our policy

The data sources of the annual migration and seasonal distribution of migratory birds involved in this paper come from published papers, which are listed in the Supplementary Data 1. We also shared the underlying dataset and code for analysis in Figshare.

Research involving human participants, their data, or biological material

Policy information about studies with human participants or human data. See also policy information about sex, gender (identity/presentation), and sexual orientation and race, ethnicity and racism.

Reporting on sex and gender	The study did not involve human research participants.
Reporting on race, ethnicity, or other socially relevant groupings	n/a
Population characteristics	n/a
Recruitment	n/a
Ethics oversight	n/a

Note that full information on the approval of the study protocol must also be provided in the manuscript.

Field-specific reporting

Please select the one below that is the best fit for your research. If you are not sure, read the appropriate sections before making your selection.

☐ Life sciences ☐ Behavioural & social sciences ☒ Ecological, evolutionary & environmental sciences

For a reference copy of the document with all sections, see nature.com/documents/nr-reporting-summary-flat.pdf

Ecological, evolutionary & environmental sciences study design

All studies must disclose on these points even when the disclosure is negative.

Study description	We use collected global dataset between 1993-2020 and reveal a strong influence of body mass on the timing and spatial patterns of bird across the full annual cycle.
Research sample	Our dataset comprised a total of tracking data of 1708 individuals from 186 species of 44 families across 19 orders, extracted from 306 papers.
Sampling strategy	For migration timing dataset, we extracted all adult birds breeding in North Hemisphere with at least one annual migration.
Data collection	The migratory birds with full annual migration data and data sources that are involved in the Supplementary Data 1.
Timing and spatial scale	Migration timings in this study were collected during 1993-2020 across the globe.
Data exclusions	We collected data for adult birds, excluding data for juveniles and subadults because birds of different ages show differences in migration timing, and excluded birds breeding in the Southern Hemisphere because they have different migration patterns than those breeding in the Northern Hemisphere. We also excluded birds breeding in the tropics as well as seabirds (e.g., penguins, petrels, pelagic gulls and terns, and auks) because their migration behaviour often lacks regular seasonal variation.
Reproducibility	The raw data of migration timings can be reproducible following Supplementary Fig. 1. The phylogeny trees of migratory birds were extracted from Jetz et al 2012 (https://birdtree.org/subsets/).
Randomization	We randomly subset 1000 phylogeny trees from BirdTree (https://birdtree.org/subsets/).
Blinding	Not applicable.

Did the study involve field work? ☐ Yes ☒ No

Reporting for specific materials, systems and methods

We require information from authors about some types of materials, experimental systems and methods used in many studies. Here, indicate whether each material, system or method listed is relevant to your study. If you are not sure if a list item applies to your research, read the appropriate section before selecting a response.

Materials & experimental systems

n/a	Involved in the study
☒	☐ Antibodies
☒	☐ Eukaryotic cell lines
☒	☐ Palaeontology and archaeology
☒	☐ Animals and other organisms
☒	☐ Clinical data
☒	☐ Dual use research of concern
☒	☐ Plants

Methods

n/a	Involved in the study
☒	☐ ChIP-seq
☒	☐ Flow cytometry
☒	☐ MRI-based neuroimaging

Plants

Seed stocks	n/a
Novel plant genotypes	n/a
Authentication	n/a
